# The role of tumor-associated macrophages in the progression, prognosis and treatment of endometrial cancer

**DOI:** 10.3389/fonc.2023.1213347

**Published:** 2023-09-22

**Authors:** Yihan Sun, Genyi Jiang, Qianhua Wu, Lei Ye, Bilan Li

**Affiliations:** ^1^ Department of Gynecology, Shanghai First Maternity and Infant Hospital, School of Medicine, Tongji University, Shanghai, China; ^2^ School of Medicine, Tongji University, Shanghai, China

**Keywords:** tumor-associated macrophages, endometrial cancer, macrophage polarization, hormones, metabolism, immunotherapy

## Abstract

Tumor-associated macrophages (TAMs) are the main immune cells in the tumor microenvironment (TME) of endometrial cancer (EC). TAMs recruitment and polarization in EC is regulated by the TME of EC, culminating in a predominantly M2-like macrophage infiltration. TAMs promote lymphatic angiogenesis through cytokine secretion, aid immune escape of EC cells by synergizing with other immune cells, and contribute to the development of EC through secretion of exosomes so as to promoting EC development. EC is a hormone- and metabolism-dependent cancer, and TAMs promote EC through interactions on estrogen receptor (ER) and metabolic factors such as the metabolism of glucose, lipids, and amino acids. In addition, we have explored the predictive significance of some TAM-related indicators for EC prognosis, and TAMs show remarkable promise as a target for EC immunotherapy.

## 1 Introduction

Endometrial cancer (EC) is an epithelial malignant tumor that occurs in the endometrium and is one of the three most common tumors of the female reproductive system. In recent years, the incidence and mortality rates of endometrial cancer have risen along with the increase in the average life expectancy of the population and changes in lifestyle habits. According to the Global Cancer Statistics 2020, EC has become the second most common tumor of the female reproductive system and the sixth most common female cancer (1). EC has even jumped to the number one position in some developed countries and cities. EC is a malignancy highly associated with hormones and metabolism, and associated risk factors including continuous estrogen exposure, diabetes, hypertension, obesity, excessive alcohol consumption and inflammation can induce the development of EC (2). According to the mechanism of occurrence and biological behavior characteristics can be divided into type I (estrogen-dependent) and type II (non-estrogen-dependent) (3). There are also the commonly used The Cancer Genome Atlas (TCGA) classification (4), which is funded by National Cancer Institute, classifying EC into four molecular subtyping: 1) POLE ultramutated 2) microsatellite-instability high(MSI-H) 3) copy-number low (CNL) 4) copy number high (CNH). And also the TransPORTEC typing (5) and PreMisE typing (6). The former is a simplified classification system of high-risk EC based on key molecular characteristics including p53-mutant, MSI, POLE proofreading-mutant and no specific molecular profile, while the latter is a partial substitution of gene sequencing by immunohistochemical methods to classify EC patients into four types.

EC is still treated with a combination of mainly surgery, selective radiotherapy and chemotherapy (7). However, for patients with advanced metastases and recurrences, it is known that there is no particularly effective treatment available, so there is an urgent need to explore new ways to treat EC (8). Immunotherapy is emerging as a new area of research and treatment for EC (9). Target selection for immunotherapy is crucial. Tumor-associated macrophages have been found to play a pivotal role in the development of cancer and are a new hot spot for recent research. In EC, their important role is also slowly being explored, and targeting tumor-associated macrophages for EC appears to be a promising option.

Tumor-associated macrophages (TAMs) are macrophages present in the tumor-associated microenvironment (TME) (10). TAMs originate from monocytes, which are specifically recruited into the tumor microenvironment through recruitment and polarized into functionally distinct TAMs under different stimuli, playing both anti-tumor and pro-tumor roles in the development of tumors.

In view of the important role of TAM in tumor development, TAMs have become a hot star in tumor immunity, especially in colorectal cancer (11) and prostate cancer (12), and have been well studied in female-related cancers such as breast cancer (13), reproductive system tumors such as ovarian cancer (14) and cervical cancer. However, studies related to endometrial cancer, which is also one of the three major tumors of the female reproductive tract, are more limited. This review summarizes the basic and clinical researches either *in vitro* or *in vivo* based on TAMs and endometrial cancer, and overviews the existing research progress on the occurrence and development of TAMs in EC, so as to provide some help for future research based on TAMs in EC.

## 2 TAMs in EC

Cancer development, progression and invasion occur in a complex and dynamic microenvironment, the growth of which is referred to as the tumor-associated microenvironment (TME). The tumor-associated microenvironment consists of various cells (immune cells, endothelial cells, fibroblasts) as well as non-cellular components (vascular network, lymphatic shutdown, cytokines, nutrients, etc.) (15). Nowadays, it is found that macrophages play an essential role in tumor development, and the macrophages in the TME are called tumor-associated macrophages (TAMs), which are the most abundant immune cells in the TME (16).

TAMs have two main origins, including tissue-resident macrophages of embryonic origin (17) and circulating monocytes of bone marrow origin (18). Embryonic-derived tissue-resident macrophages are derived from the yolk sac during the embryonic period, and embryonic stem cells gradually differentiate into tissue-specific resident macrophages that locally add value, self-renew, and have primarily pro-fibrotic functions, providing a nurturing ecological environment during the early stages of tumor progression. Bone marrow-derived circulating monocytes originate from bone marrow hematopoietic stem cells, which differentiate into monocytes that are recruited into the TME by relevant chemokines and cytokines and polarized into TAMs during tumor development, mainly playing an antigen-presenting role to drive tumor progression (19).

During tumor development, tumor cells, B cells, stromal fibroblasts, and TAMs themselves in TME secrete various cytokine, complement, and exosome components that are used to recruit monocytes to the tumor site through chemotaxis and to further polarize them into TAMs, which play a role in tumor development (20). Chemokines CCL-2/5, macrophage colony-stimulating factor (CSF-1), vascular endothelial growth factor (VEGF), and complement components such as C5a are widely recognized to have an important role in recruiting monocytes. CCl-3/4, CXCL-12, MCSF and TGF (21) have also been found to recruit monocytes in several projects.

Among them, CCL-2 and CSF-1 are well-studied cytokines. CCL-2 is mainly produced by malignant epithelial cells, and CCL-2 recruits monocytes by binding to the receptor CCR-2 expressed on monocytes, and disruption of this CCL-2/CCR-2 axis leads to a significant decrease in the number of TAMs (22). In EC, CCL-2 is regulated by the upstream LKB1 gene, an oncogene encoding a serine protein kinase that is widely expressed *in vivo*. The LKB1/AMPK/CCL-1/TAM axis leads to increased recruitment of monocytes at the tumor site, mainly some M2-like TAMs that play a pro-tumor function and promote the development of EC. Inactivation of LKB1 leads to hypophosphorylation of AMPK in epithelial cells, resulting in increased CCL-2 expression. CCL-2 acts as a key factor in the recruitment of TAMs, contributing to increased peripheral monocytes and increased monocyte recruitment in tumor TME (23). The recruitment of CSF-1 on macrophages in EC has also been demonstrated. The recruitment of CSF-1 on monocytes has been demonstrated in EC (24). By chemotactic migration assay, we found that CSF-1 secreted by EC cells can bind to CSF-1 receptors on the surface of macrophages, promote macrophage migration, and induce M2-like polarization during the subsequent polarization process, which ultimately promotes EC cell proliferation (24).

**Figure 1:**
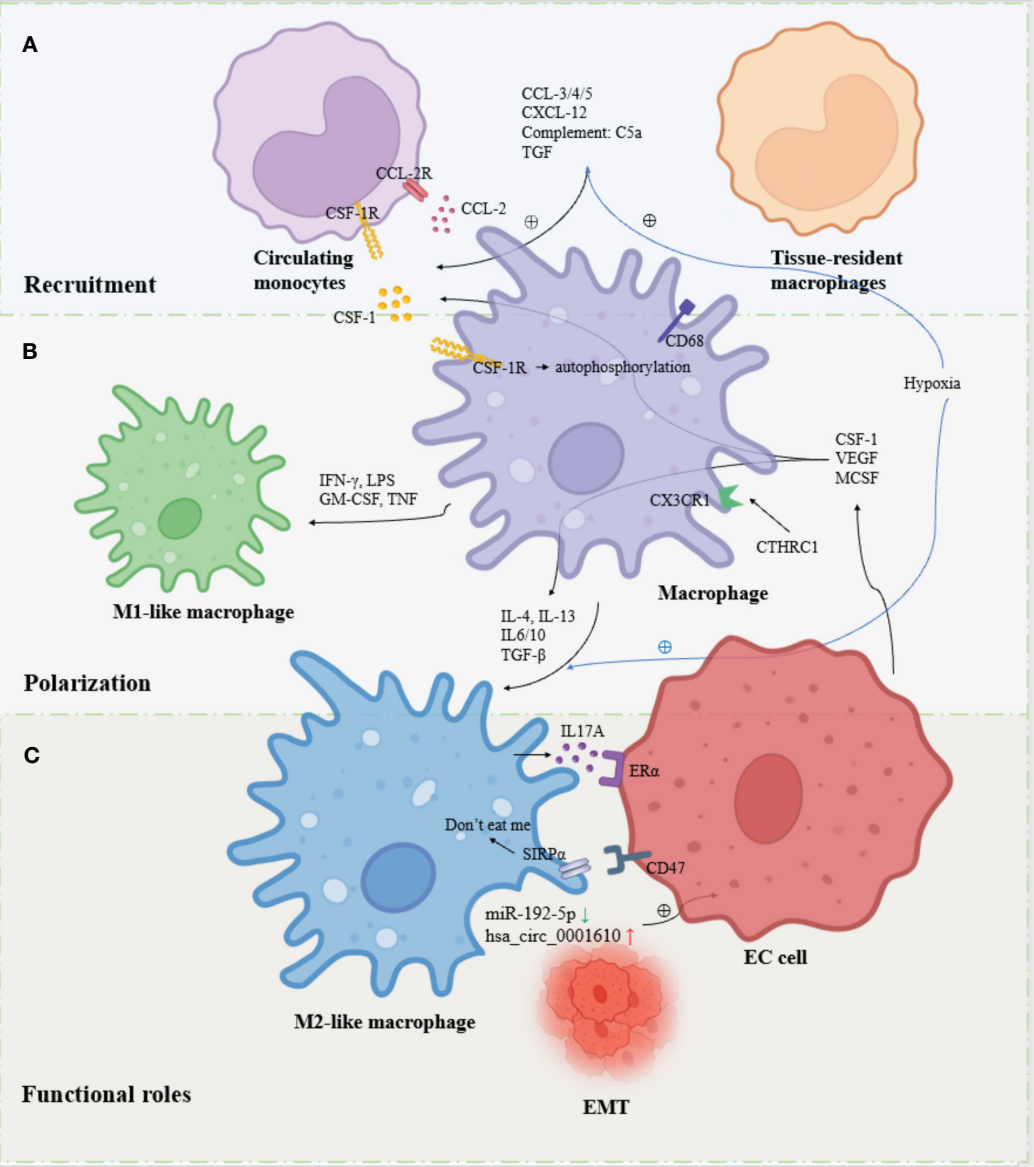
Overview of the recruitment, polarization and functional roles of TAMs in EC. **(A)** Circulating monocytes and tissue-resident macrophages are recruited to the TME and differentiate into macrophages in response to factors. **(B)** Macrophages are polarized into M1-like macrophages and M2-like macrophages in response to different signaling stimuli. **(C)**. M2-like macrophages are the major contributors to EC progression through cytokine, exosome, hormonal and metabolic pathways.

The recruitment of TAMs in EC is positively affected by CTHRC1, a secreted ECM protein found to be highly expressed in EC. overexpression of CTHRC1 activates the macrophage surface chemokine receptor CX3CR1 through the integrin β3/PI3K/Akt pathway and enhances the recruitment of M2-like TAMs, thereby promoting tumor migration and invasion (25). Some metabolic factors such as hypoxia (26) and lactate (27) and glucose enable increased recruitment of cytokines to TAMs at this site, promoting TAMs recruitment and migration of tissue-resident macrophages, thus promoting tumor development. In hypoxic conditions, tumor cells recruit TAMs by down-regulating receptors for cytokines such as CCL2, CCL5, and CSF1 (28) (**Figure 1A**).

TAMs produce different polarization states under different signals, exerting both anti-tumor and pro-tumor effects. The M1/M2 dichotomy is more commonly applied nowadays. M1-like macrophages are mainly polarized by interferon (IFN-γ) (29)produced by TH1 cells and type 1 immune response, lipopolysaccharide (LPS) produced by bacteria, granulocyte-macrophage colony-stimulating factor (GM-CSF) (30)and tumor necrosis factor (TNF), mainly TNF -α induces polarization and exerts mainly antitumor effects (31). Some other cytokines such as IL-1, IL-12 have also been shown to be involved in M1 polarization. M2-like macrophages, on the other hand, are mainly involved in tumor immunosuppression, tumor invasion, tumor growth, angiogenesis and metastasis by TH2 cells and type 2 immune responses producing chemokines IL-4, IL-13 (32) and CSF-1 secreted by tumor cells to induce polarization. Several other cytokines such as IL-6, IL-10, TGF-β, MCSF and VEGF have also been shown to be involved in M2 polarization. Although, the diversity of TAM was further uncovered with the study methodology. Based on single-cell RNA sequencing (scRNA-seq) and other single-cell analysis tools found that TAMs rarely display a true M1 or M2 phenotype, the traditional M1/M2 dichotomy does not show the diversity of TAMs well and distinguishes them from other cells. Researchers have classified more categories within the macrophage population, such as M2a, M2b and M2c (33, 34). However, some current studies still make more use of the M1/M2 dichotomy, so the traditional M1/M2 dichotomy is also followed in this article (**Figure 1B**).

CSF-1 produced by tumor cells interacts with CSF-1R, which undergoes autophosphorylation, triggers downstream signaling and determines macrophage differentiation. In endothelial carcinoma, this process is influenced by upstream CSF1R germline genetic variation. CSF-1 exhibits more significant phosphorylation and a higher proportion of M2-like macrophage polarization in TAMs with genotype CSF1R c.1085A_A (35).

Several genes upstream of endothelial carcinoma have been shown to regulate EC development through altered polarization for TAMs (33).TP53 and CDH1 are common mutated genes in type II EC. Approximately 80% of type II EC carry TP53 mutations and CDH1 inactivation. Using the TP53 and CDH1 double ablation model, it was observed that deletion of these two genes promotes the development of the tumor microenvironment by producing downstream factors of NFκB signaling, inducing macrophage polarization toward M2, and stimulating TAMs to produce chemokines, cytokines, and enzymes associated with chronic inflammation (36).

Some long non-coding RNAs (lncRNAs) have also been shown to be involved in the regulation of TAMs polarization (37). In endothelial cancer, the expression of lncRNA member NIFK-AS1 was significantly reduced compared to healthy individuals. Further studies showed that overexpression of NIFK-AS1 could inhibit the M2 polarization of TAMs by downregulating miR-146a, a microRNAs (miRNAs) that promote the M2 polarization of TAMs (38).

In addition, hypoxia also affects the M2 polarization of TAMs. Hypoxia can promote the M2 polarization of TAMs by altering the secretion of chemokines and exosomes. In EC, the promoting effect of hypoxia on the M2 polarization of TAMs has been demonstrated (39). Under hypoxic conditions, EC cells secrete more exosomes compared to normoxic conditions, in which the expression of component miRNA-21 is significantly increased, and the uptake of miRNA-21 by monocytes and the high expression and significant release of polarization-related cytokine IL-10 are observed in this process, suggesting that EC cells under hypoxic conditions can shift through the secretion of exosomes miRNA-21 to differentiate and polarize monocytes toward M2 (39).

TBBPA, a brominated flame retardant widely found in computer boards, textiles, furniture and other consumer products (40), was shown to promote migration and invasion of EC cells by promoting M2 polarization of TAMs. TBBPA-mediated binding of miR-19a to the 3′-UTR region of SOCS1, a member of the cytokine signaling family that plays a key regulatory role in macrophage polarization This leads to downregulation of SOCS1 and subsequently promotes phosphorylation of Janus kinase (JAK) and STAT6 of the signal transducer and activator of transcription(STAT) family, another key player in macrophage polarization, to promote M2 polarization of TAMs (41). This may indicate the possible upstream pathway and influencing factors of M2-like polarization of TAMs.

TAMs each express different markers depending on their polarization status (42).The main pan-macrophage marker is CD68 (43), which is widely expressed by macrophages, but is limited because it is also expressed by cells other than TAMs and cannot distinguish between specific M1 and M2 subtypes. biomarkers of M1-like macrophages include mainly CD80 and CD86. Biomarkers of M2-like macrophages are mainly CD163, CD204, CD206 (44–48), IL10, Arg-1, CCL-17, CCL-22, etc. (49).

## 3 The role of TAMs in EC progression headings

TAMs can play both antitumor and pro-tumor roles in tumor progression (19). Antitumor effects of TAMs are mainly mediated by M1-like macrophages, which produce immunologic cytotoxicity molecules (e.g., reactive oxygen species and nitriles), antitumor immune-related vasopressors (e.g., IL-12 and CXCL10), and some pro-inflammatory. The pro-tumorigenic effects of TAMs are mainly mediated by M2-like macrophages. M2-like macrophages suppress immune responses by secreting anti-inflammatory cytokines (e.g., IL-10, TGF-β) and promote angiogenesis by secreting pro-angiogenic factors (e.g., matrix metalloproteinases and VEGF), which accelerate tumor recurrence and metastasis and ultimately promote EC progression. There are many excellent reviews available on the role of TAMs in tumor progression (31), and this review will focus on the important role of TAMs in EC progression.

### 3.1 TAMs are predominantly M2-polarized in EC and play a major role in promoting EC progression

TAMs play an important role in the progression of EC. TAMs in TME of EC are mainly dominated by M2-like macrophages, so the role of TAM in EC development is dominated by the promotion of EC development, invasion, infiltration, metastasis, and immune escape. A retrospective study based on 163 EC patients found that EC had a large infiltration of M2-like macrophages and that the presence of TAMs promoted tumor aggressiveness, as evidenced by higher tumor grade, increased lymphatic vessel density, more lymphatic vessel space invasion and more lymph node metastases (50). In EC, TAMs have also been shown to be associated with myometrial invasion and vascular space invasion (51) and to mediate the immunosuppressive microenvironment in EC (16).

### 3.2 Specific upstream and downstream regulatory mechanisms of TAMs in promoting EC progression

Bioanalysis based on single cell data, a large number of TCGA RNA-seq and other tools showed that macrophage infiltration in EC is influenced by the expression of ZEB2, an important gene in epithelial mesenchymal transition, showing a positive correlation.ZEB2 is highly expressed in TAMs (16).

For the important role of EC cell invasion, M2-like macrophages can promote EC invasion progression by activating angiogenesis (52). CCL18 from TAMs upregulates KIF5B expression and promotes EMT by activating the PI3K/AKT/mTOR signaling pathway in endometrial cancer (53).

Tumor cells expressing membrane protein CD47 bind to SIRPα on the surface of TAM, which transmits “don’t eat me” signals to macrophages through downstream signaling pathways and inhibits the phagocytic ability of macrophages. It was found that CD47 was also overexpressed on the surface of EC cells, suggesting that the CD47-SIRPα signaling pathway may be an important mechanism for TAMs to mediate immune evasion of EC cells (54) (**Figure 1C**).

### 3.3 TAMs promote EC progression by interacting with other cells in the TME

TAMs can interact with other immune cells in TME such as T cells, dendritic cells, and NK cells to influence tumor development.

Regulatory T cells (Treg) are an immunosuppressive effector cell. Some results in other cancers suggest that TAMs may produce CCL20, promoting the recruitment of Treg cells into the tumor (55) while Treg cells are able to secrete IL-4, IL-13 and IL-10, which polarize macrophages toward M2 (56) and synergistically promote EC progression. The positive correlation observed between the number of TAMs and Treg cells in endometrial cancer suggests that we this interaction is also present in EC (50).

### 3.4 TAMs can promote EC progression by interacting with exosomes

Exosomes are extracellular particles between 40 and 160 nm in diameter that can be released into the pericellular environment. Currently, scientists have identified exosomes miR-192-5p and hsa_circ_0001610 interacting with TAMs to promote EC progression.

Aberrant reduced expression of miR-192-5p in TAMs- produced exosomes promotes EC development by promoting EMT and inhibiting apoptosis in EC cells. And this phenomenon could be reversed by overexpression of miR-192-5p, which inhibited EMT and promoted apoptosis in EC cells by suppressing the IRAK1/NF-κB signaling pathway (57). M2-polarized TAMs enhance the EMT of EC cells and reduce the radiosensitivity of EC cells through the exosome hsa_circ_0001610. In the exploration of its specific mechanism, it was found that hsa_circ_0001610 could down-regulate the expression of miR-139-5p through competitive binding to miR-139-5p. MiR-139-5p continues to interact with cyclin B1, an important driver of drug resistance in cancer through regulation of the cell cycle, increasing its expression and thereby reducing the radiosensitivity of EC cells. *In vivo*, the results showed increased proliferation and invasion of EC cells, inhibition of apoptosis, and decrease of G2/M cell cycle arrest. Further, this result was reversed by overexpression of miR-139-5p, suggesting the possibility of exosomes as potential targets for EC radio resistance (58). Given the crosstalk between exosomes in TAMs and EC, the composition and content exosomes may have a potentially important role in identifying EC and endometrial precancerous lesions (59) such as endometriosis (60), and the use of liquid biopsies to detect exosomes may be developed as a potential indicator for the early diagnosis and determination of prognosis of EC (**Figure 1C**).

## 4 The role of hormones in EC on TAMs

As already mentioned, EC is a malignancy highly associated with hormones. It is classified into type I and type II according to whether it is estrogen-dependent stimulation or not. Type I EC, also known as estrogen-dependent, is thought to occur in association with estrogen, possibly due to the development of endometrial hyperplasia or atypical hyperplasia followed by cancer in the presence of higher levels of estrogen over a long period of time, demonstrating the important role of hormones, especially estrogen, in the development of EC. Several studies have found that TAMs are involved in hormone-related regulation of EC. Estrogen receptor (ER) is widely expressed in infiltrating macrophages in endometriosis, ovarian cancer and other diseases. there are two main subtypes of ER, ERα and ERβ.

Related studies have shown that ERα expression in TAMs in EC is positively correlated with EC progression. Macrophage ERα agonists promote the secretion of large amounts of chemokine CCL-18 by M2-like macrophages through the ERK1/2 pathway, and CCL-18 upregulates the expression of KIF5B, thereby promoting EMT in EC through activation of the PI3K/AKT/mTOR signaling pathway and promoting aggressive progression and metastasis of EC (53). M2-like macrophages in EC induce upregulation of ERα expression by secreting the cytokine IL17A, thereby enhancing the proliferative effect of estradiol in EC cells and increasing the sensitivity of the endometrium to estrogen, thereby promoting type I EC (61). However, in contrast, a study by Tong et al. found an inverse relationship between TAMs and ERα expression in endometrial cancer tissues. They found that TAMs secreted chemokine CXCL-8, which inhibited the expression of ERα by inducing transcription factor HOXB13, and thus promoted EC invasion (62). It is interesting to note that different studies have obtained diametrically opposed results, and the effect of the role of ERα and TAMs in EC progression needs further investigation (**Figure 1C**).

## 5 The role of metabolic factors on TAMs in EC

In addition to being a hormone-dependent tumor, EC is also a metabolic disease, and among the risk factors for EC, diabetes mellitus (hyperglycemia), hypertension, and obesity all contribute to the increased incidence of endometrial cancer, which is also known clinically as the “EC triad”. Metabolic factors can also influence the development of tumors by interacting with TAMs.

TAM polarization correlates with features of glucose metabolism (63), lipid metabolism (64), and amino acid metabolism (65). M1-like macrophages show higher glucose uptake, aerobic glycolysis, and lactate production. M2-like macrophages show a predominance of lipoprotein uptake, and fatty acid oxidation (66).

In EC, it has only been observed that EC cells under hypoxic conditions promote M2 polarization of TAMs through exosome secretion (39). Although there are few studies on the influence of metabolic factors on EC progression through the TAMs pathway, the metabolic/TAMs pathway would be a good idea to investigate based on the important role of metabolic factors in endometrial tissues and even (67) EC development and the known modulation of TAMs by metabolic factors in some cancers.

## 6 Prognostic significance of TAMs for EC

TAMs by themselves can be used as prognostic indicators for EC. High TAMs often suggest poor prognostic outcomes, as evidenced by high TAMs counts suggesting shorter recurrence-free survival and overall survival (50)

It was found that women with LKB1-deficient EC had elevated levels of the pro-TAMs recruitment chemokine CCL-2 in peripheral blood serum, suggesting that CCL-2 produced by the tumor-bearing uterus can enter the circulation and that serum CCL-2 may be used as a potential circulating biomarker to monitor tumor progression or predict progression risk, but this is still at an early stage and not supported by relevant data (23).

## 7 Prospects of TAMs as therapeutic targets for EC

Considering the important role of TAMs in the development of EC, TAMs can be a very promising target for EC therapy. The main ideas of targeting TAMs for EC treatment include depleting M2-like macrophages, targeting related signaling pathways such as recruitment and accumulation, and promoting M2 to M1 reeducate.

Targeting the recruitment and accumulation of TAMs is a good therapeutic target. Drugs targeting macrophage related markers such as CSF-1, IFN-γ, VEGF, chemokines, and cell surface antigens are being gradually put into trials, and are one of the future research focuses of tumor immunotherapy targeting macrophages (19). In EC, Interfering with CSF1-CSF1R, CCL2-CCR2 has been shown to functionally deplete TAMs by targeting recruitment and survival of TAMs within tumors (68, 69). In EC, given the positive role of CSF-1 in recruiting monocytes and promoting M2 polarization, CSF-1R blockers targeting its receptor could block the recruitment of TAMs in EC by targeting CSF-1 to reduce the number of TAMs and slow down EC progression (24). Moreover, by finding that macrophages in the population possessing the immunoglobulin-like structural domain 4 of CSF-1R within the amino acid change from histidine to arginine, the CSF1Rc.1085A>G gene variant, exhibited resistance to CSF-1 stimulation and were more susceptible to CSF-1R inhibitors in terms of receptor phosphorylation and endocytosis, suggesting that our CSF- 1Rc.1085A>G gene variant could be used as a potential predictive biomarker for predicting the efficacy of targeting CSF-1R signaling for cancer therapy (35). What’s more, targeting the CCL-2 axis such as LKB1 has also been identified as a potent inhibitor of EC (23).

CD40 receptors are a member of the TNF receptor family and are expressed on antigen presenting cells, including TAMs. activation of CD40 plays an important role in antitumor immunity. targeting of TAMs by CD40 agonists is associated with re-education of immunosuppressive TAMs into cytotoxic effectors, ultimately leading to immune surveillance and reduction of tumor growth (70). In EC, CD40 activation of M1-like macrophages resulted in effective tumor growth inhibition but failed to reverse the proliferative effects of M2-like macrophages (71).

CD47 is expressed in normal and cancer cells and sends “don’t eat me” signals to macrophages, which is an important way for normal cells to protect themselves from phagocytosis and a pathway for immune escape of tumor cells. Anti-CD47 antibodies targeting tumor cell surface membrane proteins can improve the phagocytosis of M2 TAMs on EC cells by blocking the EC CD47-SIRPα immunosuppressive pathway. Inhibition of EC development by re-education of M2-like macrophages (54).

The repolarization of M2-like macrophages may become an effective therapeutic modality. Huang et al. simulated the EC tumor microenvironment by constructing a disintegrable supramolecular gelatin hydrogel model, which was able to help IFN-γ reprogram M2-like macrophages to M1-like macrophages, in which progress the reduction of VEGF secretion was observed. The reprogrammed M2-like macrophages showed reduced migration of cancer cells *in vitro* and inhibition of tumor cell growth *in vivo*. It may serve as an effective tool for future drug delivery and treatment (72).

## 8 Summary and prospect

TAMs play an important role in the progression of EC. TAMs in EC are recruited from monocytes and are polarized in different directions by different cytokines and other factors in the tumor microenvironment, giving rise to M1-like macrophages, which exhibit mainly antitumor effects, and M2-like macrophages, which are pro-tumor. The infiltration of M2-like macrophages is predominant in EC. M2-like macrophages promote tumor angiogenesis, EMT, immune evasion and metastasis in EC by secreting cytokines, synergizing with other immune cells, and through exosomal effects. TAMs also regulate EC progression by interacting with hormonal conditions such as ER and metabolic conditions such as oxygen saturation. TAMs can also serve as a marker to detect EC prognosis. Finally, given their essential function in promoting EC progression, TAMs are also a very promising target for EC immunotherapy in the future.

Compared with other cancers such as colorectal cancer, breast cancer and ovarian cancer, where TAMs and tumor mechanisms are well researched, some of the existing researches on EC are mostly in the demonstration of the relevance of TAMs to promote the development of EC, but lack more in-depth mechanism studies. Furthermore, as EC is a hormonal and metabolic-dependent cancer, the influence of estrogen and glucose, lipids, and other factors is undeniable, and it is also a very promising entry point for how TAMs interact with hormonal and metabolic factors to influence the development of EC. It is also very promising to take TAMs as the entry point to explore the changes of tumor immune microenvironment after treatment or recurrence and metastasis of endometrial cancer or whether the different microenvironment represented by TAMs has a predictive effect on the progression, recurrence, treatment and prognosis of endometrial cancer. In conclusion, research on the role of TAMs in EC progression, prognosis, and treatment is still emerging, and given the important role of TAMs in other gynecologic tumors, research on their role in EC must be promising.

## Author contributions

YS wrote the main manuscript text and prepared the figures and tables. BL provided suggestions on the framework of this review and corrected the mistakes. QW, GJ and LY helped with the literature review during writing. All authors contributed to the article and approved the submitted version.
